# Relationship between Desiccation Tolerance and Biofilm Formation in Shiga Toxin-Producing *Escherichia coli*

**DOI:** 10.3390/microorganisms12020243

**Published:** 2024-01-24

**Authors:** Muhammad Qasim Javed, Igor Kovalchuk, Dmytro Yevtushenko, Xianqin Yang, Kim Stanford

**Affiliations:** 1 Department of Biological Sciences, University of Lethbridge, Lethbridge, AB T1K 3M4, Canada; mq.javed@uleth.ca (M.Q.J.); igor.kovalchuk@uleth.ca (I.K.); dmytro.yevtushenko@uleth.ca (D.Y.); 2 Agriculture and Agri-Food Canada, Lacombe, AB T4L 1V7, Canada; xianqin.yang@agr.gc.ca

**Keywords:** *Escherichia coli*, STEC, biofilm, desiccation, relative humidity, bacterial survival, stainless steel, food processing facilities

## Abstract

Shiga toxin-producing *Escherichia coli* (STEC) is a major concern in the food industry and requires effective control measures to prevent foodborne illnesses. Previous studies have demonstrated increased difficulty in the control of biofilm-forming STEC. Desiccation, achieved through osmotic stress and water removal, has emerged as a potential antimicrobial hurdle. This study focused on 254 genetically diverse *E. coli* strains collected from cattle, carcass hides, hide-off carcasses, and processing equipment. Of these, 141 (55.51%) were STEC and 113 (44.48%) were generic *E. coli*. The biofilm-forming capabilities of these isolates were assessed, and their desiccation tolerance was investigated to understand the relationships between growth temperature, relative humidity (RH), and bacterial survival. Only 28% of the STEC isolates had the ability to form biofilms, compared to 60% of the generic *E. coli*. Stainless steel surfaces were exposed to different combinations of temperature (0 °C or 35 °C) and relative humidity (75% or 100%), and the bacterial attachment and survival rates were measured over 72 h and compared to controls. The results revealed that all the strains exposed to 75% relative humidity (RH) at any temperature had reduced growth (*p* < 0.001). In contrast, 35 °C and 100% RH supported bacterial proliferation, except for isolates forming the strongest biofilms. The ability of *E. coli* to form a biofilm did not impact growth reduction at 75% RH. Therefore, desiccation treatment at 75% RH at temperatures of 0 °C or 35 °C holds promise as a novel antimicrobial hurdle for the removal of biofilm-forming *E. coli* from challenging-to-clean surfaces and equipment within food processing facilities.

## 1. Introduction

Foodborne contamination and the subsequent risk of foodborne illness depend on various factors, including worker practices, food storage, food preparation surfaces, waste management and food hygiene practices [1,2]. Pathogens such as bacteria, viruses, fungi, and parasites can contaminate food products throughout the production line, from production and manufacturing to distribution, preparation, and final consumption [2]. Among these pathogens, bacteria, particularly those belonging to the Enterobacteriaceae family, pose a significant threat to the food industry [3,4]. In particular, the *Salmonella* and Shiga toxin-producing *E. coli* (STEC) genera are responsible for severe foodborne infections [5,6]. Foodborne infections caused by pathogens can lead to severe harm and even death, especially among immunocompromised individuals, the elderly, and young children [7]. In the case of STEC, infections are particularly concerning due to their global outbreaks and pathogenicity [5]. Cattle serve as the main reservoir for STEC, with various serotypes linked to human sickness. Pathogenic bacteria, including STEC, can attach to surfaces and form biofilms, which present a challenge in food manufacturing settings as biofilms confer extreme resistance against sanitizers, antibiotics, and other external factors [8,9,10]. Over 90% of bacteria exist as a biofilm on various biotic and abiotic surfaces, including stainless steel, rubber, plastic, silicon, and glass in food manufacturing settings [10]. The proportion of STEC strains forming biofilms is relatively low, but STEC able to form extremely strong biofilms exists [11]. Effective control methods are required to prevent the formation of biofilms, which can lead to cross-contamination and compromise food safety [8,12]. Various chemical, physical, and mechanical methods have been explored to control biofilm formation but are either expensive or of limited efficacy in some situations [13]. These methods include the use of essential oils [14], enzymes [15,16], biosurfactants [17], photosensitization [13], ultrasonic waves [18,19,20], and electric fields [21,22].

Salt is widely used in the food industry due to its preservative and antibacterial properties [23]. It disrupts the osmotic balance within bacterial cells, leading to osmotic dehydration when bacterial cells are immersed in hypertonic solutions [24]. This dehydration process can partially dewater the cells and impact their cellular components and their survival [24]. The effect of salt on biofilm formation and bacterial survival was judged to be of particular interest [25], but to date there have been few studies evaluating desiccation for the control of biofilms. Numerous studies have reported the prolonged survival of *E. coli* O157:H7 on stainless steel surfaces, even at low temperatures [26,27,28]. As stainless steel surfaces are commonly used in food processors, this demonstrates the ability of STEC to persist and increases the risk of cross-contamination from contaminated surfaces to food items. By examining the resistance of *E. coli* isolates to desiccation and temperature variations, this study aimed to enhance our understanding of biofilm control strategies and the factors influencing *E. coli* survival and cross-contamination risks in food processing environments.

## 2. Materials and Methods

### 2.1. Selection of Isolates

Based on the ability of *E. coli* to form biofilm determined in a previous study [11], a total of 254 strains were selected from both generic *E. coli* and STEC (Table 1). Briefly, overnight cultures were diluted by combining 50 μL with 5 mL of fresh Luria-Betani medium (LB, Oxoid Ltd., Basingstoke, Hampshire, UK). A 160 μL portion of the diluted inoculum was added to duplicate wells of a round-bottom 96-well microtiter plate. Each plate included duplicate blank wells with LB medium as a negative control, and positive controls featured a known strong biofilm-forming isolate (O121:H23). After incubation at 15 °C for 4 d, microplate absorbance at 570 nm was measured using a microplate reader and biofilm formation was categorized as described in Table 1. The *E. coli* was originally isolated from cattle and their environment, cattle carcasses, or processing equipment as described in a previous study [29]. The STEC isolates were balanced as much as possible across different biofilm-forming classes.

### 2.2. Preparation of Relative Humidity Tubes

Based on methodology from a previous study [28], salt (NaCl; Sigma Aldrich, St. Louis, MO, USA) was dissolved in distilled water to achieve RH 75% (375 g/1 L), while distilled water alone was used for RH 100%. The solutions were autoclaved and dispensed into 50 mL centrifuge tubes, with each tube containing 800 µL of the respective solution. To ensure accuracy and replicate the experiment, four tubes were prepared for each RH level (75% and 100%). All replicate tubes were processed side by side at the same time on the same day as described below. Within each RH, duplicate tubes were prepared for each equilibration temperature, with tubes incubated at 0 °C or 35 °C for a period of 3 weeks.

### 2.3. Coupon Preparation

Food-grade stainless steel coupons (5.0 cm × 2.0 cm, grade 304, no. 4 finish) were soaked in a detergent solution (Tergazyme, Alconox Inc., New York, NY, USA) overnight. They were subsequently washed in distilled water, followed by 70% ethanol, and left to air dry, following a previously described protocol [28]. The coupons were then autoclaved and used in the subsequent experiments.

### 2.4. Bacterial Culture Conditions

For revitalization, the bacterial culture was inoculated from glycerol stocks onto MacConkey agar plates (Dalynn, Calgary, AB, Canada) and incubated at 35 °C for 24 h. Subsequently, a single colony was selected for subculture in 10 mL of ½ strength brain heart infusion broth (BHIB) (Oxoid). The culture was placed in a shaking incubator at 35 °C and 80 rpm for 16–18 h, resulting in a bacterial suspension with a concentration of 5–6 log CFU/cm^2^, which was then used for inoculating the coupons. These culture conditions were the same as used in a previous study [29] for the same strains of *E. coli* where concentrations of colonies were confirmed by enumeration on MacConkey agar.

### 2.5. Inoculation and Incubation of Stainless Steel Coupons

The coupons were inoculated by adding 50 µL of bacterial culture to one half of the coupons in individual sterile petri dishes. The petri dishes were left in a biosafety cabinet for 10 to 15 min to allow excess moisture to be absorbed. Subsequently, the equilibrating tubes were opened one at a time to prevent contamination and loss of moisture. Using sterile forceps, the inoculated coupons were carefully placed in the tubes, ensuring they did not contact the liquid, and the lids were tightly sealed. The tubes were then incubated at the same temperatures used for equilibration (0 °C or 35 °C) for a duration of approximately 72 h.

### 2.6. Assessment of Bacterial Counts

Freshly prepared 50 mL centrifuge tubes containing 30 mL 0.1% (*w*/*v*) pH 7.2 peptone water (Oxoid) were utilized, and the inoculated coupons with bacterial suspension were added to the tubes. Glass beads (0.5 mm) were autoclaved and pre-weighed, and 3 g was added to the tubes prior to vortexing the tubes at maximum speed for 1 min. For each sample, 10-fold serial dilutions were performed, and 1 mL of culture was filtered through a 0.45 µm membrane filter (Millipore S-Pak Type HA, 47 mm gridded, Millipore Sigma, Oakville, ON, Canada) using vacuum filtration prior to plating the filter on Lactose Monensin Glucuronate Agar (LMG Agar, Oxoid), a selective media for *E. coli*. The agar plates were incubated at 35 °C for 20–24 h, and the resulting blue colonies were counted. These plates served as the control for bacterial survival without exposure to any stress environment. The same procedures were used to assess bacterial numbers on the coupons incubated for 72 h at either 0 or 35 °C and these were compared to the controls.

### 2.7. DNA Extraction and PCR Confirmation

To confirm colonies were *E. coli*, DNA was extracted by lysing cells using TE buffer as previously described [30]. The amplification of bacterial DNA through PCR was performed utilizing a set of primers for the *uidA* gene (Table 2) [31], which resulted in the amplification of DNA fragments comprising 166 base pairs. Cycling conditions consisted of an initial denaturation at 95 °C for 5 min, followed by 40 cycles, and each cycle consisted of a 30 s denaturation step at 95 °C, a 60 s annealing step at 60 °C, and an extension period at 50 °C for one minute. The amplified DNA products were made visible using the QIAxcel system (QIAXcel Advanced, Qiagen, Toronto, ON, Canada). For all PCR reactions, a strain of *E. coli* O157:H7, EDL933, was used as a positive control, while a strain of *Salmonella enterica* was used as a negative control.

### 2.8. Statistical Analysis

Statistical analyses were performed using SAS Version 9.4 (SAS Institute Inc., Cary, NC, USA) in a mixed effects model comparison. Fixed effects included temperature, humidity, and biofilm-forming class in a factorial design. Percentage reduction was the response variable and was calculated by following equation:

$$\mathrm{Percentage}\;\mathrm{reduction}=1-\left(\frac{\mathrm{CFU}\;\mathrm{Treatment}}{\mathrm{CFU}\;\mathrm{Control}}\right)\times 100$$

The normality of the response variable was confirmed using Proc Univariate, with *p* > 0.05 for each test of normality employed. Additionally, to compare the percentage reduction between STEC and generic *E. coli*, we employed RStudio (R Core Team, 2023 https://www.R-project.org, accessed on 10 January 2024). In this analysis, we performed a *t*-test to determine 95% confidence intervals using a Bonferroni correction to avoid inflating Type 1 error. Trends were determined if 0.05 < *p* < 0.1, with significance at *p* < 0.05. The findings were visually represented using ggplot within RStudio, highlighting the distinctions between STEC and generic *E. coli*, the impact of biofilm class, and the effects of different treatments.

## 3. Results

### 3.1. Colony Morphology

**Figure 1:**
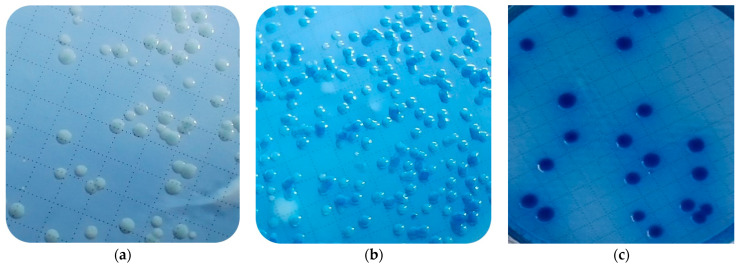
Unexpected colony morphology after growth of *E. coli* on LMG agar after 72 h including (**a**) entirely colorless colonies and (**b**) partial blue colonies in comparison to (**c**) expected blue colonies.

The strains retrieved from frozen glycerol stocks were streaked on MacConkey agar plates (Dalynn), where they exhibited pink shiny textured colonies with well-defined margins. After incubation on LMG agar, colonies that lacked the expected blue coloration (Figure 1c) were occasionally noted. These included colorless colonies (Figure 1a) and partially blue-colored colonies (Figure 1b). Regardless of these variations in morphology, PCR amplification with *uidA* gene primers confirmed all as *E. coli*.

### 3.2. Effects of Temperature and Relative Humidity

**Figure 2:**
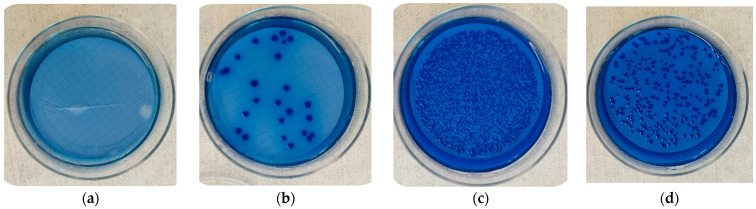
Growth of *E. coli* strains on LMG agar under different conditions over 72 h: (**a**) Lack of growth at 35 °C and 75% RH, (**b**) Reduced growth when exposed to 0 °C and 75% RH, (**c**) Increased growth when exposed to 35 °C and 100% RH, and (**d**) Bacterial colonies from control without any treatment.

In our investigation of desiccation stress, we subjected the *E. coli* strains to various combinations of temperature and relative humidity, exposing them to 75% or 100% RH at either 0 °C or 35 °C for approximately 72 h. Both temperature and relative humidity influenced (*p* < 0.05) bacterial growth compared to the controls. For the 10^−4^ dilution, a mean of 51 CFU ± 18 CFU was observed for the controls (Figure 2d). All the strains exposed to 35 °C or 0 °C and 75% RH had reduced growth (*p* < 0.05) (Figure 2a,b, respectively). Also, when exposed to 0 °C and 100% RH, bacterial growth was negligible (*p* < 0.001). Conversely, the combination of 35 °C and 100% RH provided optimal conditions that supported bacterial proliferation, which on average led to significantly increased growth compared to the controls (*p* < 0.001; Figure 2c).

### 3.3. Effects of Biofilm-Producing Ability

**Figure 3:**
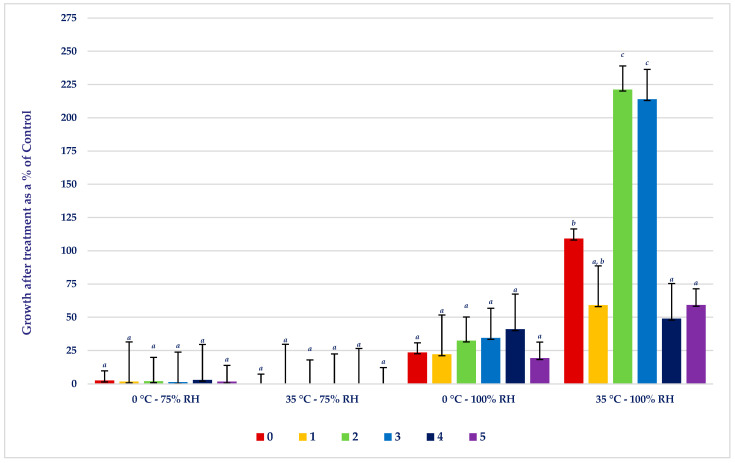
Influence of biofilm class on growth as a percentage of control after treatments. Comparison of desiccation tolerance between different biofilm formation categories. Biofilm classes are defined as 0 = non-biofilm former, 1 = weak biofilm, 2 = moderate biofilm, 3 = strong biofilm, 4 = very strong biofilm, and 5 = extremely strong biofilm. *^a^*^,*b*,*c*^ Means with different superscripts within treatment combinations differ (*p* < 0.05).

We observed that the biofilm-forming class of the isolate had a notable impact on the growth of isolates at 35 °C and 100% RH. Specifically, under what we expected to be optimal growth conditions, strains that were not capable of forming biofilm (class 0), moderate biofilm formers (class 2), and strong biofilm formers (class 3) had increased cell proliferation (>100% of that of controls), while isolates capable of forming the strongest biofilms (class 4 and class 5) as well as weak biofilm formers (class 1) had reduced cell growth, <100% of that of the controls (Figure 3). However, at 75% RH, the growth of all the strains was reduced compared to that of the controls and was not affected by the ability of the isolates to form biofilm.

### 3.4. Effects of Desiccation on STEC as Compared to Generic E. coli

**Figure 4:**
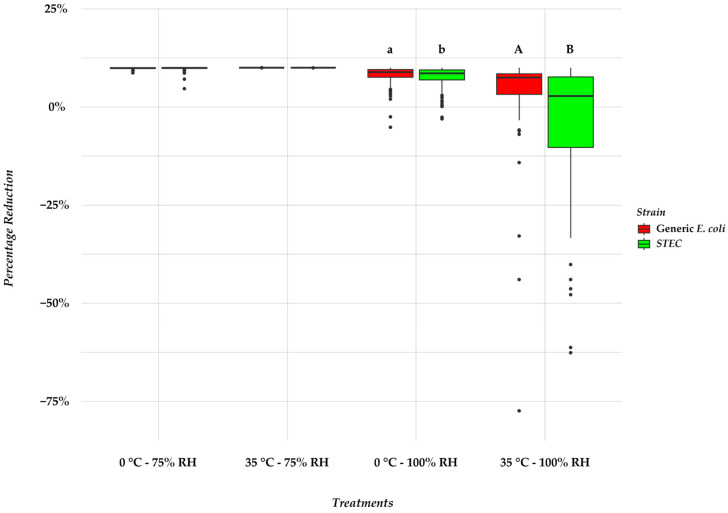
Box plot representation of percentage reduction values for various treatments in biofilm formation. The data are categorized by STEC and generic strains, denoted by different colors and each point indicates one isolate. The box plot illustrates the distribution of percentage reduction values within each treatment, providing insights into the variability and central tendencies of the data. Means within treatment combinations with different superscripts differ. ^a,b^ Generic and STEC strains tended to differ in percentage reduction (*p* < 0.1). ^A,B^ Generic and STEC strains differed in percentage reduction (*p* < 0.001). Negative percentage reductions are indicative of cell growth.

In our study, we compared bacterial reductions between STEC and generic *E. coli* across four different conditions (0 °C + 75% RH, 35 °C + 75% RH, 0 °C + 100% RH, 35 °C + 100% RH). We found no difference (*p* = 0.36) in reduction between the STEC and generic isolates at 75% RH and 0 °C. Similarly, at 35 °C and 75% RH, the STEC and generic *E. coli* reductions were almost identical (*p* = 0.5). However, at 0 °C and 100% RH, the STEC exhibited a trend (*p* = 0.1) to a lower reduction compared to the generic *E. coli* of approximately 5.05%. Under optimal conditions, i.e., 35 °C and 100% RH, the STEC isolates had a lower reduction compared to the generic isolates of approximately 85.82% (*p* < 0.001; Figure 4).

## 4. Discussion

### 4.1. Atypical Colony Morphology

During our study, several *E. coli* isolates had colonies with unexpected morphologies when cultured on LMG agar after exposure to desiccation stress. Initially, the unexpected differences in colony appearance raised concerns of potential contamination. However, we later confirmed these colonies as *E. coli* through PCR amplification. LMG agar contains lactose as a carbon source and the *lac* operon is one of the best-known gene regulatory circuits, exemplifying how bacteria adapt their metabolism to nutritional conditions [32]. Stressors that disrupt the *lac* operon could have led to the changes in colony color. Fluctuations in temperature can impact lactose utilization by impacting the enzymatic activity of β-galactosidase as demonstrated by Fujikawa [33], where *Pantoea agglomerans* could produce blue pigment only at temperatures of ≥10 °C. Also, the activity and stability of enzymes involved in pigment synthesis are directly affected by the pH of the growth media. A pH fluctuation can be induced by metabolite accumulation, nutrition intake, oxygen availability, and organic acid outflow [33]. The color shift by several isolates in our study was potentially caused by stress-induced gene regulation, genetic variability, and/or adaptive responses.

### 4.2. Effects of Temperature and Relative Humidity

Pathogenic bacteria often encounter various abiotic stressors, such as drying, temperature fluctuations, oxidative conditions, pH changes, and osmotic pressures, among others [8]. These stress factors exert selective pressures on the resilience and virulence of these bacteria, leading to immediate effects on shorter timescales and evolutionary changes over longer periods [34]. Numerous investigations have explored the impact of these abiotic stresses on bacterial characteristics [35,36,37,38,39]. The ability of bacteria to withstand desiccation varies among different species. In general, gram-positive bacteria exhibit greater tolerance to dry conditions compared to gram-negative bacteria [40,41,42]. This study revealed that, even though the numbers of *E. coli* decreased on steel surfaces under dry conditions (75% relative humidity) or in the cold (0 °C), certain cells persisted, while others exhibited robust growth under the conditions of 35 °C and 100% RH for over 72 h. Desiccation tolerance has been previously investigated in *E. coli*, alongside three other bacterial cultures [43]. These researchers found that *E. coli* O157:H7 displayed the lowest resistance to desiccation compared to all the other strains. The greatest reduction in O157:H7 viability was observed when it was subjected to 24 h of desiccation at a relative humidity of 33% and pH levels of 4, 5 and 7 [43]. Furthermore, Hwang et al. [44] assessed the reduction in *E. coli* O157:H7 when exposed to temperatures of 22 °C and relative humidity ranging from 80% to 85% for a period of 3 to 7 d. These researchers observed a reduction in bacterial count between 0 and 3.5 log_10_ CFU/g in sausages [44]. This finding is relevant to our current study because it underscores the relationship between lower relative humidity and a decrease in bacterial count.

Nissen and Holck [45] reported that *E. coli* O157:H7 in dry sausages, which had a pH of 4.8 and water activity (aw) 0.89 exhibited greater inactivation when stored at 20 °C compared to 4 °C. Similarly, Chikthimmah and Knabel [46] revealed that bologna inoculated with 7.5 log_10_ CFU of *E. coli* O157:H7, decreased contamination more effectively when stored at 13 °C rather than 3.6 °C. Interestingly, our study contradicts these, as we observed better reductions in bacterial counts regardless of temperature when the relative humidity was lower and generally reduced growth at 0 °C as compared to 35 °C. Desiccation stresses likely differ in a food matrix as compared to the stainless steel coupons used in the present study and differences among studies could be result of variations in the food matrix, pH, water activity, and strain-specific characteristics of *E. coli* O157:H7. The matrix where evaluations occur undoubtedly affects experimental results. *E. coli* grows rapidly in soil temperatures above 30 °C but cells also have a higher mortality rate in warmer conditions (>30 °C) compared to colder temperatures (<15 °C) [47,48]. Another study indicated that *E. coli* can endure prolonged periods at temperatures lower than those typically found in host organisms [49] but in our study both the low and optimal temperatures could reduce bacterial growth provided relative humidity was also reduced.

### 4.3. Effects of Biofilm-Forming Class

As all the strains during the current study showed reduced growth compared to the controls after incubation at 75% RH, there were no significant differences in desiccation sensitivity among the classes of biofilm-formers. However, at 35 °C and 100% relative humidity, the extent to which the isolates were able to form biofilm influenced bacterial growth. Possibly, the initial growth of the most extreme biofilm formers was rapid and used the nutrients available, resulting in cell death during the 72 h incubation. Previous studies [50,51] determined that biofilm formation is most rapid at higher temperatures and the biofilm composition changes at low temperatures. When *E. coli* was cultivated in culture media with 0% or 1% NaCl supplementation, biofilm formation was detected, although the presence of 3.5% or 5% NaCl completely inhibited the development of the biofilm [52]. Other investigations support the notion that osmotic stress can induce biofilm production in various microorganisms, including *Staphylococcus epidermidis*, *Clostridium ljungdahlii*, and *Candida albicans* [53,54,55]. Perhaps extreme biofilm producers are more sensitive to osmotic stress than other *E. coli* strains. Weak biofilm formers (class 1) also showed reduced growth compared to the controls at 35 °C and 100% RH, possibly due to strain-related variation. The factors promoting biofilm formation in *E. coli* are poorly understood and are worthy of further study.

Another critical factor to consider pertains to oxygen availability. Changes in temperature and humidity can impact the levels of dissolved oxygen within the environment and a previous study hypothesized that elevated temperature at low oxygen availability increases biofilm formation [56]. Like many other bacterial species, *E. coli* adapts its growth behavior in response to oxygen availability. As the tubes were sealed during the 72 h incubation, the initial rapid growth of the extreme biofilm formers may have reduced oxygen levels and impeded overall bacterial growth [56]. Moreover, bacteria, including *E. coli*, employ quorum sensing and signaling molecules to communicate and coordinate behaviors like biofilm formation. Previous studies [57,58] determined that autoinducer 1 and autoinducer 2 are related to biofilm formation and regulation, respectively, in extreme environments in *Acinetobacter baumannii*, *Pseudomonas aeruginosa*, and *Bifidobacterium longum*. Temperature and humidity can potentially affect the production and reception of these signaling molecules, subsequently impacting the timing and intensity of biofilm development and cellular survival [59]. These relationships reinforce the need to consider multiple elements when devising strategies to regulate biofilm formation, with potential implications in various fields, such as healthcare and food safety.

### 4.4. Effects of Desiccation on STEC vs. Generic E. coli

The survival rates of the STEC strains were equal to those of the generic *E. coli* in our study under 75% RH. This observation contrasts with earlier research, which had indicated lower survival rates for non-pathogenic *E. coli*. during desiccation [60] but aligned with another study using similar methodology on stainless steel coupons where complete inactivation was observed in generic *E. coli* and O157 when exposed to 75% RH and 35 °C [28]. Despite not being widely considered resistant to desiccation, *E. coli* O157:H7 has demonstrated greater survival abilities compared to generic *E. coli* during drying [61,62,63,64]. The ability of STEC to survive in dry foods has led to outbreaks caused by STEC contamination since the mid-1990s [64]. The improved growth at optimal conditions (35 °C and 100% RH) for STEC noted in the present study was likely due to fewer STEC strains being able to form extremely strong biofilms compared to generic *E. coli*, with only 2 STEC extreme biofilm formers vs. 48 of the generic *E. coli*.

### 4.5. Is Desiccation a Potentially Useful Antimicrobial Hurdle for Beef Slaughter Plants?

Common advice to the food industry is to keep the production environment less humid (75% RH) to prevent bacterial growth [65]. However, contrary to earlier findings of decreased survival at 85% RH and greater survival of *E. coli* at RH < 85% [65], our study observed better bacterial inhibition at 75% as compared to 100% RH. Other studies evaluated desiccation in dry foods such as confectionary products and chocolates [60] as opposed to our studies with stainless steel coupons. Better surface attachment [66], an abundance of organic residues in desiccated food [67], and a protective matrix in food such as sugar crystals [68,69] could be possible reasons for improved bacterial survival in dry foods as compared to stainless steel under a desiccated environment.

While the meat industry typically operates at around 80% RH [70], intermittent periods of higher humidity during cleaning routines could contribute to microbial survival. Even though this study indicated that *E. coli* survival was reduced at 75% RH after 72 h, maintaining such precise humidity levels would likely be impractical for the food industry. A more feasible approach would be to limit periods of elevated humidity at temperatures > 0 °C, as these conditions could enhance STEC survival, growth, and biofilm formation. Also, the environment of meat processing facilities would not likely have the same restrictive effect on extreme biofilm formers seen in the present study as nutrients and oxygen would both be abundant.

Previous research indicated that STEC survival rates in food were higher in dry conditions (aw 0.5 to 0.6) at 4–15 °C compared to 20–35 °C [27,59,71], a trend shared by other bacteria [42]. In our study, survival was better at 35 °C compared to 0 °C, with an average of 24% more growth at 35 °C and 100% RH, which is likely reflective of differences in the survival of *E. coli* in food as compared to stainless steel. This study’s findings indicate desiccation at 75% RH was effective for growth inhibition regardless of temperature, although inhibition was most pronounced at 0 °C. Accordingly, desiccation may be a valuable strategy for mitigating the prevalence of enteric pathogens on surfaces within the context of beef processing. The utility of NaCl sprays, independently and in tandem with more aggressive compounds such as sodium hypochlorite, n-alkyl dimethyl ethylbenzyl ammonium chlorides, and peroxyacetic acid is worthy of additional study.

## 5. Conclusions

Our study explored the impact of desiccation stress on *E. coli*, uncovering unexpected results. Atypical colony morphologies initially raised concerns, but PCR confirmation verified them as *E. coli*. Our research showed that 75% RH yields better bacterial inhibition than 100% RH, in contrast to some other studies evaluating desiccation effects in food matrixes. The investigation showed that biofilm formation involves complex dynamics, with bacterial growth being influenced by temperature and humidity. Survival of the STEC and generic *E. coli* was equivalent at 75% RH but differed most markedly at 35 °C and 100% RH, likely due to the reduced biofilm-forming ability of STEC. Overall, our research indicates that desiccation at 75% humidity might be a promising approach for mitigating enteric pathogens on surfaces in beef processing. However, additional studies are necessary to determine when to apply salt sprays in conjunction with traditional sanitizers.

## Figures and Tables

**Table 1:** Biofilm-forming classes of *E. coli* isolates as determined by optical density (OD).

Biofilm Class ^1^	Generic *E. coli*	STEC ^2^
0, non-biofilm former	45	92
1, weak	4	4
2, moderate	2	20
3, strong	4	10
4, very strong	10	0
5, extremely strong	48	2

^1^ Biofilm-forming class is as follows: non-biofilm former, x < ODc; weak < ODc < x < 2 x ODc; moderate, 2 x ODc < x < 4 x ODc; strong, 4 x ODc < x < 8 x ODc; very strong biofilm formers, 8 x ODc < x < 16 x ODc; and extremely strong, 16 x ODc < x. ^2^ Shiga toxin-producing *E. coli* isolates. x = OD of two independent replicates for each isolate. ODc = three times the standard deviation of OD of negative control plus average OD of negative control.

**Table 2:** Forward and reverse primers for *uidA* gene for generic PCR amplification of *E. coli* STEC.

Primer Set	Sequence
*uidA*	F: 5′ TGGTAATTACCGACGAAAACGGC 3′
	R: 5′ ACGCGTGGTTACAGTCTTGCG 3′

## Data Availability

All relevant data were presented within this manuscript. Other data are available upon request.
